# Role of Telemedicine and Telehealth in Public Healthcare Sector: A Narrative Review

**DOI:** 10.7759/cureus.69102

**Published:** 2024-09-10

**Authors:** Vaibhavi Shende, Vasant Wagh

**Affiliations:** 1 Department of Community Medicine, Datta Meghe Institute of Higher Education and Research, Wardha, IND

**Keywords:** barriers and challenges, digital health, recent advance technologies, telehealth, telemedicine, telemonitoring

## Abstract

Clinicians, researchers in health services, and other experts have been investigating how to improve healthcare using advanced computer and telecommunication technology for more than 30 years. Adequate medical facilities are still lacking in many places of the world. In these kinds of situations, technology can be quite helpful in expanding healthcare access to rural locations and offering better care at a lower cost. The delivery of healthcare is changing dramatically because of telemedicine and telehealth, particularly in terms of improving access to care. This paper aims to provide an update on the history, background, applications, benefits, barriers, and challenges of these recent technologies. This review article also covers the healthcare conditions of rural as well as urban communities. Furthermore, the implications of technologies used and improvement in the health status of an individual are also discussed. During the COVID-19 epidemic, telehealth quickly gained popularity, bringing to light a number of issues. Effective primary medical networks are crucial, as the COVID-19 pandemic highlighted the need for improving public health responses during crises and revealed the existing fragmentation in healthcare delivery systems.

## Introduction and background

Clinicians, researchers in health services, and other professionals have been investigating how to improve healthcare delivery through the use of advanced computer and telecommunication technology for more than 30 years. Telemedicine is at the centre of these initiatives, which integrates conventional and cutting-edge information technologies [1]. Researchers in health services and medicine have dedicated over 30 years to exploring how computer and advanced communications technology can improve patient care. On the more traditional side, this includes the established use of radio to link emergency medical professionals with hospitals and the telephone for patient-physician consultations [1]. The speed at which digital technologies have improved is staggering, but very often the capabilities of health-care providers and payers to deliver and use these changes have not kept up with them. The technologies are ready for deployment, however, the features of rurality and remoteness in which these new tools need to be implemented will have special characteristics that should be taken into account [2]. Telemedicine reduces healthcare costs, thereby overcoming barriers to accessing high-quality care and potentially encouraging more people to seek healthcare services [3]. The aim of the study is to guide the general information about the telehealth and telemedicine in various key points. This review focuses on the emergence of telehealth and telemedicine, their use, and also about the recent advances technology in healthcare sector. It not only explains the barriers and challenges of telehealth and telemedicine but also includes the recent advances in technology as well as the COVID-19 pandemic era. The purpose of this review is to explore the impact of telemedicine on healthcare accessibility. By examining the existing literature, case studies, and empirical evidence, this review aims to elucidate how telemedicine can address barriers to healthcare access and improve the overall accessibility of healthcare services. In addition, this review will identify challenges and limitations associated with the adoption of telemedicine and telehealth and how to maximize the potential benefits of telemedicine and telehealth in promoting healthcare accessibility.

History and definition

In the early 1900s, the initial documented instance of telemedicine involved the transmission of electrocardiograms over telephone lines. About 50 years ago, telemedicine was dismissed due to its cumbersome, unreliable, and expensive nature. The first telemedicine service in India was pioneered by Apollo Hospital in Andhra Pradesh, establishing a connection with Apollo Hospital in Chennai. Successful examples of telemedicine implementation in India include mammography services at Sri Ganga Ram Hospital in Delhi and oncology services at the Regional Cancer Center in Trivandrum [4]. A new generation of healthcare platforms, telehealth, and telemedicine allow patients and hospital staff to communicate while also providing smartphone access to a range of health information. Telehealth services can address any medical issue or concern, regardless of its severity or specialty, by enabling patients to consult with healthcare providers remotely through technologies such as video calls or online platforms. While health information can assist us in selecting the most appropriate course of treatment, our health cannot be purchased in the same manner as medications. During the 1970s, the term "telemedicine" was coined, literally meaning "remote recovery" [5]. In the early 1900s, modern telemedicine had its origins in the Netherlands when heart rhythms were first transmitted over telephone lines. This development was followed in the 1920s by the transmission of medical consultations via radio to centers across Europe. By the 1940s, telephone connections were used to send radiographic images between Pennsylvanian cities [5].

Telemedicine

Since the word "telemedicine" is derived from the Latin term *mederi*, which means "to heal," and the Greek word *tele*, which means "distance," it literally means "distance healing". The World Health Organization describes telemedicine as “the delivery of health care services, where distance is a critical factor, by all health care professionals using information and communication technologies for the exchange of valid information for diagnosis, treatment, and prevention of disease and injuries, research and evaluation, and for the continuing education of healthcare providers, all in the interests of advancing the health of individuals and their communities [6].

Telehealth

The International Organization for Standardization's definition of telehealth is the "use of telecommunication techniques for the purpose of providing telemedicine, medical education, and health education over a distance" [6].

## Review

Methodology

An automated search was performed across PubMed, Google Scholar, and Web of Science, as well as references found through an online search of databases to identify articles for potential inclusion in this review, using search terms like “telemedicine”, “telehealth”, “recent advance technologies”, “public health sector”, and their synonyms. Various filters, including full text and free full text, were utilized during the search process. After filtering the results by full free-text article availability and articles from the year 2014 till 2024, we obtained 190 records identified through the database search using the search engines. Sixty-five studies were searched from PubMed, 49 from Google Scholar, 69 from Web of Science, and seven from other websites. After screening the title and abstract, out of the 100 studies that were screened, 60 studies were initially evaluated for eligibility. Of these, 27 studies were removed because of reasons like insufficient details, only abstract available, out of scope, and some studies having limited rigor. Out of 60 studies, which were assessed for eligibility, 33 were analyzed in this review.

Discussion

Through the utilization of communication technology, telemedicine would provide medical details about patients to health professionals. Telemedicine also grants doctors access to other medical practitioners who may have information pertaining their patients. Remote monitoring technology can be used for home postoperative monitoring or to shorten the hospitalization stay of the patient after the surgery [7]. A vast range of multidimensional service groups, patient kinds, specialist types, techniques, and places are included in telehealth. The use of digital technology to facilitate rapid access to medical expertise and knowledge is known as telehealth [7]. With social separation emerging as a critical component in the battle against the COVID-19 epidemic, some medical facilities have closed and stopped treating patients in an effort to protect them from infection, drastically changing the way care is provided. The telephone-based services, including telehealth, are regarded as an acceptable and even preferable method of service delivery by clients of various behavioural health treatment programs [8].

Because of their remote locations, lack of onsite educators, travel distance, time constraints, and other factors, rural and remote physicians frequently struggle to acquire high-quality education. Obtaining schooling in isolated and rural areas can pose difficulties for medical professionals. Information and communication technology (ICT) and medical research are combined in the growing discipline of telemedicine, which has many uses in administration, teaching, and training in the healthcare industry. It holds a great deal of promise to address the difficulties of providing healthcare to isolated and rural locations. It might be as easy as two medical professionals talking over the phone about a patient's medical issues and requesting advice, or it can be as complicated as sending electronic medical records including medical information, diagnostic test results (such as ECG), radiological pictures, etc. [9].

Telemedicine's Use in Public Health

*Epidemiological surveillance*: It can provide important information about population health assessment as well as fresh perspectives on geographic variation and gradients in illness incidence and prevalence. Additionally, it supports the planning of interventions as well as the evaluation of different methods of intervention and their efficacy (systems of spatial information) the assessment of the spread of vector-borne disease can be improved by using spatial-temporal modeling to analyze climate, environmental factors, and disease transmission using geographic information system (GIS), which offers the fundamental architecture and analytical resources for this purpose. In this context, methods for remote sensing have lately been employed [10].

*Interactive health education and illness prevention*: It has the ability to communicate with both individuals and the general public. For people who live in isolated places, it can offer simple access. It encourages domiciliary care and self-care behaviors. Many people who reside in remote areas might benefit from self-management of health conditions, which also supplements the current healthcare system. It has the potential to be a highly useful tool for assessing and keeping track of healthcare services [10].

Telemedicine represents the cutting-edge of healthcare, accelerating the processes of diagnosis and therapeutic care. As technology advances, virtual health emerges as the next significant development in healthcare, appealing to both practitioners and patients alike [11]. As a triage technique that could shorten wait times and lower patient loads in emergency rooms (ERs), telemedicine has potential. After radiographs were sent over 24 miles from West Chester to Philadelphia via a teleradiology system, telemedicine was first mentioned in 1950. It quickly gained traction and is now recognized as a standard component of healthcare practice [12]. The introduction of healthcare services by every medical professional using technology for communications and information to share trustworthy data for the identification, treatment, and avoidance of illnesses and injuries is known as telehealth. Its uses include ongoing education for healthcare professionals as well as monitoring and assessment, all with the goal of contributing to the good health of people and society as a whole, a goal in which distance plays a critical role. In order to provide effective health services, medical professionals and patients alike must find a way to close the gap caused by inaccessibility and the incapacity to make in-person visits [13]. The most recent advancements and trends in population and public health informatics are examined in this article. It is slightly different from the yearbook monograph series' earlier revisions. The recently developed public health technology vision and infrastructure aim at the alignment of information systems, aims, goals, and consequences across the frequently distinct fields of both public wellness and population health and the growing use of social determinants of health (SDH). It is divided into three thematic areas which are: the newly emerging infrastructure and vision for public health informatics; the alignment of informatics objectives, goals, and outcomes across the frequently distinct fields of population health and public health; and the growing use of SDH data by SDH informatics professionals in both public and population health [14].

Population health management is made easier by telemedicine, which allows for proactive outreach to underprivileged groups, health trend monitoring, and the implementation of focused interventions to address common health conditions. This has resulted in variations in the way telehealth is done, specific to both how and what types of teleplaces are established. Research is integral to the development of guidelines, and we now have a greater than ever evidence base in telehealth that allows us develop evidence-based standards. To evaluate the awareness, utilization, and impact of the completed American Telemedicine Association (ATA) standards and guidelines on the telemedicine industry, it's important to note that guidelines are primarily designed to enhance patient safety. Respondents generally report a high level of awareness about the availability of these guidelines and their practical applications. The main uses identified are program development (55%), staff training (53%), and clinical practice guidelines/discussion meetings. These proportions highlight the importance of guidelines to payers and regulators, who are increasingly incorporating them into regulations and policies. Overall, these guidelines facilitate a better understanding of the development of practice guidelines for telemedicine. When 109 users were asked for responses on what they knew about the suggested telehealth guidelines, the responses were (in percentage) ATA 75%, professional association 42%, individual organizational guideline 31%, federal agency recommendation 27%, state regulation requirement/recommendation, 21%. in this case, 15 users reported that they were aware of the payers/Medicare reimbursement information related to coverage requirements/ restrictions or guidance before claims are filed/sent in for service (the bill does have conditions under which it is covered vs not; payers responded accordingly based on questions asked). Among the survey respondents, the majority (close to 25%) reported their organizations use in-house and/or professional association/society guidelines as well as telehealth-specific ATA best practices [15]. Another example is the research that looked into how patients' propensity to enroll in clinical trials for cancer is impacted by decentralization tools and remote technology, which are intended to reduce the time and travel difficulties involved with participation. The results indicated that the use of remote technology and other decentralization tools, which reduce the need for travel to trial sites, was linked to an increase in patients' self-reported likelihood of participating in cancer trials [16]. 

A study was done to explore telehealth in a broad sense and included technology models for clinical use, education and training of health care professionals and patients, and preventive and primary care services. It also included telehealth used for a variety of disease states and rural populations. Other notable benefits included decreased direct and indirect costs to the patient (travel cost and time) and health care service provider (staffing), lower onsite health care resource utilization, improved physician recruitment and retention, improved access to care, and increased education and training of patients and health care professionals. A recent study focused the the cost and potential to improve outcomes and access to care. Health technology interventions play a crucial role in rural healthcare by reducing staffing and travel expenses and enhancing residents' access to care, including specialized services, which would otherwise be inaccessible in remote areas. In any setting, implementing new health technologies requires careful evaluation of acceptability and feasibility. This analysis shows that underserved communities and rural populations may successfully implement telehealth, express satisfaction with its interventions, and find them to be convenient and effective [17].

Benefits and Challenges of Telehealth and Telemedicine

Because specialized care is frequently concentrated in urban areas, rural populations living in remote locations without internet access may be unable to benefit from virtual communication [4]. The drawbacks of telehealth interventions included encountering unfamiliar providers during tele-visits and encountering technical obstacles such poor Wi-Fi coverage and connectivity problems in remote areas. Several studies have found no statistically significant differences in the efficient outcomes of telehealth and regular visits [17]. Critics of telehealth, however, point out some prospective disadvantages, such as the inability to conduct comprehensive physical examinations, potential technical difficulties, security problems, and regulatory difficulties. They argue that telehealth could compromise continuity of care by being impersonal and lacking the comprehensive patient history and physical examination that traditional in-person visits provide, which are crucial for an accurate diagnosis and effective treatment [18]. In this narrative review, the role of telehealth and telemedicine in today's healthcare landscape is explored, detailing its benefits and challenges which are shown in Table 1 [19].

**Table 1:** Benefits and challenges of telehealth and telemedicine [19]. HIPAA: Health Insurance Portability and Accountability Act

	Benefits	Challenges
Telehealth	Cost-effectiveness - reduced travel, time, and expenses.	Excessive use or improper use of care - unintentional overconsumption of healthcare resources stemming from unnecessary visits.
	Improved access - reaching remote communities. Providing telemonitoring for clinicians in deserving regions.	Exacerbating current inequalities in healthcare access - inequalities in internet access and technology adoption among rural communities and ethnic minority groups.
	Enhancing emergency preparedness - swift mobilization of resources during public health emergencies and natural disasters.	Patient information security - concerns regarding HIPAA compliance in telehealth platforms and medical devices.
	Reduced mismatch between supply and demand - tackle physician shortages through a remote work approach.	
Telemedicine	Enhanced availability of information.	Deterioration in the relationship between healthcare professionals and patients.
	Delivery of care that was previously unattainable.	Disruption in the collaboration among healthcare professionals.
	Better access to services and increased delivery of care.	Problems with the quality of health information.
	Advancement in professional education.	Organizational and bureaucratic challenges.
	Improved quality control in screening programs.	
	Reduction in healthcare expenses.	

Telemedicine faces significant barriers that hinder its widespread adoption and effectiveness, necessitating thoughtful consideration and strategic interventions for resolution. Infrastructure and access issues are among the main problems, particularly in underserved and rural areas. These places frequently don't have enough access to digital gadgets and high-speed internet, which impedes the widespread utilization of telemedicine. Telemedicine encounters additional complexities due to regulatory hurdles, including diverse state regulations that govern telehealth practices and may limit physicians' ability to practice across state lines based on licensure requirements. Communication obstacles further complicate matters and many telemedicine platforms fall short in providing specialized features to accommodate healthcare interactions for individuals who are visually challenged or hearing impaired. Furthermore, there is a shortage of educational resources for patients, which particularly affects those with language barriers and low literacy levels, exacerbating their access to telemedicine services [20].

Preserving the confidentiality of personal information transmitted and stored through digital devices is a major concern in telemedicine. A significant factor contributing to privacy issues in telehealth is the inadequate understanding among healthcare professionals about safeguarding patient information while using medical equipment. Hence, it is essential for medical providers to receive thorough training and demonstrate competence in remote patient care management [21].

In a study done in New Jersey, the aim was to investigate technology engagement among low-income individuals of color residing in Newark. The findings revealed that around 20% of people in this under-resourced community did not have access to essential technologies required for effective telehealth service deployment. The timing of the study provided an unexpected opportunity to compare experiences and attitudes towards telehealth across two different regulatory environments [22]. The main drawbacks of telemedicine that can be anticipated include: a potential disruption in the patient-health professional relationship; concerns about the quality of health information; and challenges related to organizational and bureaucratic processes [23].

The primary drawbacks of telemedicine that can be anticipated include the following: The study investigated the evolving concept of therapeutic relational connection (TRC) within telehealth, focusing on analyzing its dynamics in interactions between healthcare providers and patients. The results showed the essential characteristics of TRC in telehealth include the provider's capacity to assess patient concerns, interpersonal communication skills, cultural sensitivity, mutual trust and respect, presence, empathy, and relationship-building. Antecedents to TRC encompass clinical presence, suitable environment, proficiency in technology use by both patients and providers, effective verbal and nonverbal communication, along with an understanding of community and cultural contexts [24]. A study was conducted to investigate the outcome measures utilized to assess the efficacy of telehealth in emergency departments located in rural and remote areas. Additionally, the study aimed to analyze how these outcome measures were applied and understood within the clinical setting. The findings indicated that telehealth utilization in rural and remote emergency departments (EDs) effectively improved or maintained clinical effectiveness, ensured appropriate care processes, and, in specific contexts, enhanced the speed of care. Additionally, it promoted favorable patterns of service utilization [25].

There are few points that summarize the current state of telehealth implementation in India, including the upcoming steps to its implementation. It is anticipated that the Personal Data Protection (PDP) Bill, which is presently being considered by Parliament, will soon be passed and offer strict standards for implementation and policy. Initiatives to increase healthcare professionals' capabilities and patient awareness campaigns need to be pursued. The digital gap should shrink in India with the arrival of 5G connectivity. Also, encouraging digital health and health data literacy throughout the healthcare education system is crucial and the National Digital Health Mission is well positioned to help digital health services be adopted more easily [26].

COVID-19 Pandemic and Digital Technologies

The rise of telemedicine is one of the unusual benefits of the COVID-19 pandemic [27]. Following a prompt installation of telehealth services in a community behavioral health organization in light of the COVID-19 epidemic, Bhowmik et al. evaluated the feasibility and acceptability of the services. The results of this study indicate that telehealth, including services delivered via telephone, is a well-received and often favored mode of service delivery for clients experiencing severe mental illness [8]. To gather the available data on the utilization of telehealth facilities in India throughout the COVID-19 epidemic, an analysis was conducted. The results show that the crisis and the lockdown that followed had a significant effect on the nation's healthcare system [28].

Lack of governmental support and inadequate knowledge of the uses and advantages of telemedicine are two of the main obstacles noted. This scoping review also acts as a call to action to increase public awareness of the possible benefits that telemedicine can provide in addressing various issues related to the COVID-19 epidemic and healthcare delivery [29]. Enhanced comprehension of how patients and providers perceive telehealth is essential for widespread adoption and enhanced healthcare access. According to the study's findings, telehealth is positively seen by both remote patients and providers, suggesting that using telehealth techniques could increase the value of medical services in remote areas [30].

Recent Advancements in Telehealth and Telemedicine

One of the most significant upcoming advancements in telemedicine is remote patient monitoring (RPM). RPM enables patients to use a device that sends health information to their phone or tablet, helping them keep track of their health (27). However, as people live longer, the population is getting older, which puts many nations' socioeconomic structures at jeopardy because of the rising expenses of providing healthcare and senior citizen well-being. With the help of sophisticated communication and information technologies, wearable medical and environmental sensors, actuators, and smart home automation, health and wellness of senior citizens can be continuously and remotely monitored at a lower cost [31].

A study showed that video telehealth enhances healthcare access for individuals with diabetes by providing a convenient method to receive care without needing to arrange transportation or take time off work. Additionally, this approach helps lower a patient's environmental footprint by decreasing the need for travel [32]. An investigation into the quick development of well-established telehealth procedures in pediatric cardiology and pediatrics was carried out. In order to preserve pediatric care, although digitally, the lockdown, worldwide pandemic, and social estrangement measures accelerated the adoption of clever modifications and transitions. Technology for remote cardiac monitoring is developing quickly as a result of improvements in internet connectivity, mobile handheld devices, and artificially intelligent technology [33].

## Conclusions

The review emphasizes the vital importance of telehealth and telemedicine in enhancing access to healthcare. However, telemedicine was set aside about 50 years ago because of its cumbersome, unreliable, and expensive characteristics. The first telemedicine service in India was pioneered in Andhra Pradesh. After the pandemic era, there has been a rise in the use of telehealth and telemedicine, which was found to be very useful, especially to the rural community and for people of low socioeconomic status, remote areas, and where medical facilities are minimal. This technology also plays an important role in various surgeries. At the same time, technology companies should keep innovating to create user-friendly telemedicine platforms that address the needs of both providers and patients.

Various benefits and drawbacks are also mentioned in this study. One advantage of telemedicine is its capacity to improve healthcare access for people, especially those who have trouble getting to medical facilities. Telehealth was once limited to isolated or rural locations, but it is currently being used more widely to improve access to care and increase the regional coverage of healthcare services. As technology progresses, telehealth is receiving increasing approval and acceptance, proving its efficiency and effectiveness in improving healthcare access and outcomes. However, challenges remain in rural communities, including poverty, lack of knowledge, and limited awareness, which can hinder the widespread adoption of telehealth and telemedicine.
